# Estimating unobserved SARS-CoV-2 infections in the United States

**DOI:** 10.1073/pnas.2005476117

**Published:** 2020-08-21

**Authors:** T. Alex Perkins, Sean M. Cavany, Sean M. Moore, Rachel J. Oidtman, Anita Lerch, Marya Poterek

**Affiliations:** ^a^Department of Biological Sciences, University of Notre Dame, Notre Dame, IN 46556;; ^b^Eck Institute for Global Health, University of Notre Dame, Notre Dame, IN 46556

**Keywords:** coronavirus, emerging infectious disease, mathematical modeling, public health surveillance, silent transmission

## Abstract

By March 2020, COVID-19 led to thousands of deaths and disrupted economic activity worldwide. As a result of narrow case definitions and limited capacity for testing, the number of unobserved severe acute respiratory syndrome coronavirus 2 (SARS-CoV-2) infections during its initial invasion of the United States remains unknown. We developed an approach for estimating the number of unobserved infections based on data that are commonly available shortly after the emergence of a new infectious disease. The logic of our approach is, in essence, that there are bounds on the amount of exponential growth of new infections that can occur during the first few weeks after imported cases start appearing. Applying that logic to data on imported cases and local deaths in the United States through 12 March, we estimated that 108,689 (95% posterior predictive interval [95% PPI]: 1,023 to 14,182,310) infections occurred in the United States by this date. By comparing the model’s predictions of symptomatic infections with local cases reported over time, we obtained daily estimates of the proportion of symptomatic infections detected by surveillance. This revealed that detection of symptomatic infections decreased throughout February as exponential growth of infections outpaced increases in testing. Between 24 February and 12 March, we estimated an increase in detection of symptomatic infections, which was strongly correlated (median: 0.98; 95% PPI: 0.66 to 0.98) with increases in testing. These results suggest that testing was a major limiting factor in assessing the extent of SARS-CoV-2 transmission during its initial invasion of the United States.

## Significance

In early 2020, delays in availability of diagnostic testing for COVID-19 prompted questions about the extent of unobserved community transmission in the United States. We quantified unobserved infections in the United States during this time using a stochastic transmission model. Although precision of our estimates is limited, we conclude that many more thousands of people were infected than were reported as cases by the time a national emergency was declared and that fewer than 10% of locally acquired, symptomatic infections in the United States may have been detected over a period of a month. This gap in surveillance during a critical phase of the epidemic resulted in a large, unobserved reservoir of infection in the United States by early March.

Severe acute respiratory syndrome coronavirus 2 (SARS-CoV-2) is a newly emerged coronavirus that is causing a global pandemic (1). The unprecedented spread of SARS-CoV-2 owes to its high transmissibility (2), presymptomatic transmission (3), and transmission by asymptomatic infections (4). An appreciable fraction of infections is asymptomatic (5), and many others result in mild symptoms that could be mistaken for other respiratory illnesses (6). These factors point to a potentially large reservoir of unobserved infections (7), especially in settings where capacity to test for SARS-CoV-2 has been limited (8). The United States is one such country in which limited testing was a major concern, particularly as imported cases and then local cases increased over time in early 2020 (9). Until 27 February, testing criteria in the United States were limited to close contacts of confirmed cases and those with recent travel to China (9). This means that any local infections resulting from an unobserved imported infection would have gone unnoticed. Community transmission occurred without notice while testing was still being rolled out (10, 11), albeit to an unknown extent.

Our goal was to estimate the extent of community transmission of SARS-CoV-2 in the United States that occurred prior to its widespread recognition. Unlike other countries where testing and containment measures were pursued aggressively (12, 13), rollout of testing in the United States was slow (9), and widespread social-distancing measures did not go into effect until several weeks after the first reported case (14, 15). Understanding the extent of community transmission has major implications for the effectiveness of different options for control (16) and for anticipating the trajectory and impact of the pandemic (17).

## Results

To estimate the extent of community transmission of SARS-CoV-2 in the United States, we used a stochastic simulation model that combined importation and local transmission processes. We informed model parameters with estimates from epidemiological studies of those parameters, where available (Table 1), and estimated values of two unknown parameters by fitting our model to data on local reported deaths in the United States (18). To model importation, we simulated observed and unobserved imported infections based on the number and timing of imported cases reported in the United States (19) and assumptions about the proportion of different infection outcomes (5, 20). To model local transmission, we used a branching process model informed by estimates of the reproduction number from a meta-analysis (21) and of the serial interval from a study in China (22). To relate our model’s predictions to US data on reported cases and deaths, we also simulated the timing of symptom onset (22), case reporting (18), and death (23) for simulated infections for which those outcomes occurred.

**Table 1.** Model parameters

Parameter	Baseline (alternatives)	Distribution	Reference/reason
Reproduction number, *R* [95% UI]	2.7 [1.6, 3.9]	Negative binomial (with *k*)	Davies et al. (21)
Dispersion, *k* [95% UI]	0.58 [0.35, 1.18] (0.1 [0.04, 0.2], 1000)	Negative binomial (with *R*)	Bi et al. (24) [Endo et al. (25), Poisson like]
Asymptomatic proportion, σ [95% UI]	0.432 [0.322, 0.547] (0.178 [0.155, 0.202], 0.74 [0.70, 0.78])	Beta	Lavezzo et al. (20) [Mizumoto et al. (5), Emery et al. (26)]
CFR	0.0138 [0.0123, 0.0153] (0.0086 [0.0072, 0.0103], 0.0565 [0.0550, 0.0581])	Beta	Verity et al. (23) [Deng et al. (27) outside Hubei, Deng et al. (27) overall]
Generation interval [meanlog, sdlog]	[1.51, 0.493] ([1.39, 0.568], [1.92, 0.432])	Log normal	Zhang et al. (22) [Nishiura et al. (28), Li et al. (29)]
Incubation period [shape, scale]	(2.03 [1.42, 2.64], 5.84 [4.74, 6.94]) ([1.24, 5.38], [2.45, 6.26])	Weibull	Zhang et al. (22) [Guan et al. (30), Lauer et al. (31)]
Delay in reporting following symptom onset [shape, rate]	[3.43, 0.572] ([1.72, 0.572], [5.15, 0.572])	Gamma	MIDAS Network (19) (50% lower or higher shape parameter)
Period from symptom onset to death [meanlog, sdlog]	[2.81, 0.370] ([2.19, 0.501], [2.88, 0.472])	Log normal	Verity et al. (23) [Mizumoto et al. (5) time from hospitalization to death as plausible lower bound, Wu et al. (32)]
Proportions of symptomatic imported infections detected, ρ_travel_	0.387 [0.154–0.870]	Calibrated	This is the calibrated estimate in the baseline analysis; it is recalibrated in each sensitivity analysis scenario
Relative infectiousness of asymptomatic infections, α	0.602 [0.0460–0.981]	Calibrated	This is the calibrated estimate in the baseline analysis; it is recalibrated in each sensitivity analysis scenario

All time periods are given in days; 95% UI refers to the 95% uncertainty interval.

By 12 March, there were a total of 1,514 reported cases and 39 reported deaths that resulted from local transmission of SARS-CoV-2 in the United States. We used that information to estimate the probability of detecting imported symptomatic infections, ρ_travel_, by seeding our model with imported infections, simulating local transmission, and comparing simulated and reported local deaths. In our baseline analysis, this resulted in a median estimate of ρ_travel_ = 0.45 (95% posterior predictive interval [95% PPI]: 0.04 to 0.97). This wide uncertainty reflects several factors, including that the simulations used to estimate ρ_travel_ incorporated uncertainty in eight model parameters informed by other studies. Along with ρ_travel_, the one other parameter that we estimated (relative infectiousness of asymptomatic infections, α) also displayed wide uncertainty (median: 0.55; 95% PPI: 0.04 to 0.98) and was positively correlated (Pearson’s correlation: 0.40) with ρ_travel_ (*SI Appendix*, Fig. S4). The fact that calibration of our model yielded such broad estimates of ρ_travel_ and α was neither surprising, given that these estimates were informed by only a single data point (39 cumulative deaths) nor concerning given that their estimation was secondary to our primary objective of estimating unobserved infections.

**Fig. 1.**
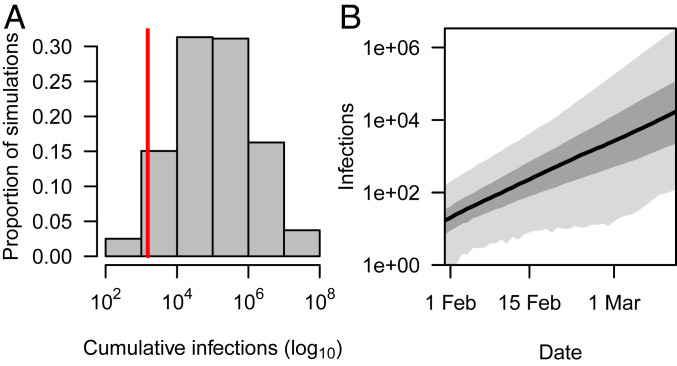
Local infections of SARS-CoV-2 in the United States by 12 March. These results derive from our baseline analysis and show (*A*) cumulative and (*B*) daily incidence of local infections. In *A*, the red line shows the cumulative number of reported cases by 12 March (1,514), indicating that the cumulative number of infections exceeded the cumulative number of reported cases in 95.7% of simulations. The model’s predictions of cumulative infections, which were informed by data on cumulative deaths (33) and parameter estimates from the literature (Table 1), exceeded 10,000 in 82.5% of simulations and 100,000 in 51.3% of simulations. In *B*, the model’s prediction of daily incidence of infection was 100/d or less in early February but grew exponentially to thousands per day by 12 March. The black line shows the median, dark gray shading shows the interquartile range, and light gray shading shows the 95% PPI.

Simulating from 1 January, we obtained 108,689 (95% PPI: 1,023 to 14,182,310) local infections cumulatively in the United States by 12 March (Fig. 1*A*). Due to the exponential growth posited by our model, 17,122 (95% PPI: 120 to 3,366,705) local infections were predicted to have occurred on 12 March alone (Fig. 1*B*). This wide uncertainty reflects a combination of stochasticity inherent to early epidemic growth, particularly for a pathogen with heterogeneous transmission (24), and uncertainty about model parameters. Estimates of cumulative local infections in our sensitivity analysis were reasonably robust to most alternative parameterizations (*SI Appendix*, *Supplementary Text* and Fig. S5), with the exception of a scenario in which a long serial interval ruled out the possibility of more than a million infections. Overall, this suggests that uncertainty in our results was driven primarily by parameter uncertainty already accounted for in our baseline analysis. With values ranging from 1.6 to 3.9 in its 95% uncertainty interval, uncertainty in *R* seemed to account for much of the uncertainty in our estimate of cumulative local infections (*SI Appendix*, Fig. S6).

**Fig. 2.**
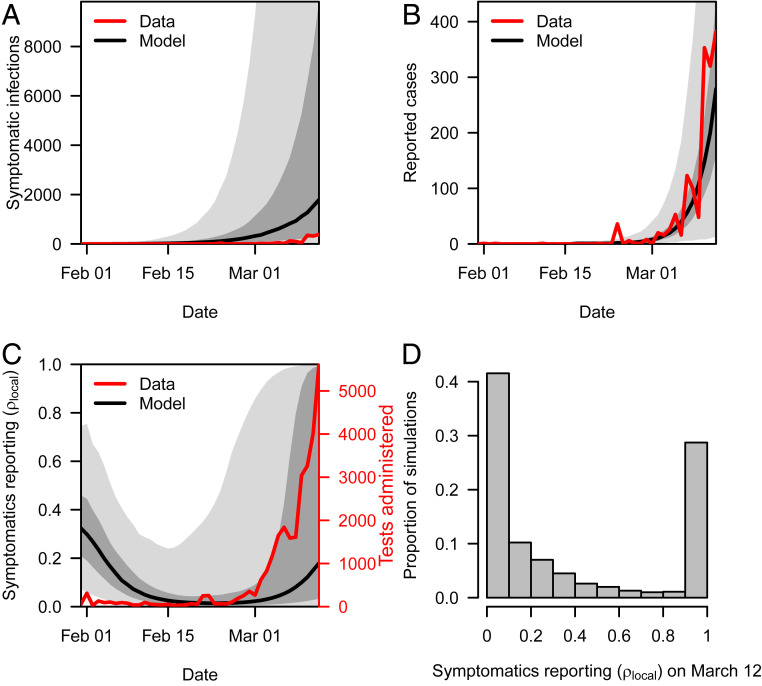
Comparison of symptomatic infections and reported cases. (*A*) Local symptomatic infections predicted under the baseline analysis (black and gray) increased exponentially and at much greater magnitude than reported cases (red). (*B*) To reconcile these differences, we estimated a time-varying probability of detecting symptomatic infections, ρ_local_, which yielded model predictions of reported cases (black and gray) consistent with daily reported cases (red). (*C*) An initial decline in ρ_local_ (black and gray) resulted from exponential growth of symptomatic infections (*A*) outpacing relatively constant testing (red) in early February. A later increase in ρ_local_ is consistent with a sharp increase in testing through late February and early March. (*D*) By 12 March, most symptomatic infections were still likely going undetected, despite increases in testing. Our analysis resulted in some estimates of ρ_local_ approaching one on 12 March, due to a portion of simulations with fewer symptomatic infections than reported cases that day. This was a consequence of the model being informed by data on cumulative deaths only and not reported cases. Black lines show the median, dark gray shading shows the interquartile range, and light gray shading shows the 95% PPI.

Had we performed a simple extrapolation of reported cases and deaths based on ρ_travel_, our estimate of cumulative local infections by 12 March would have been only 6,050 (95% PPI: 2,752 to 19,986). This suggests that detection of local infections was less sensitive than detection of imported infections. To estimate the probability of detecting local symptomatic infections, ρ_local_, we compared our model’s predictions of symptomatic infections with local case reports on a daily basis (Fig. 2*A*). Over the course of February, daily estimates of ρ_local_ decreased from our uniform prior down to a low of 0.015 (95% PPI: 0.001 to 0.465) on 24 February, as increases in simulated local infections outpaced newly reported local cases (Fig. 2*B*). Our results indicate that detection of symptomatic infections was below 10% for around a month (median: 31 d; 95% PPI: 0 to 42 d) when containment still might have been feasible. As testing increased in March (Fig. 2*B*, red), so too did reported cases (Fig. 2*C*, red) and daily estimates of ρ_local_ (Fig. 2*B*, black). By 12 March, we estimated ρ_local_ to be 0.176 (95% PPI: 0.002 to 0.999) (Fig. 2*D*). Uncertainty in the degree to which ρ_local_ rose in early March was driven to a great extent by uncertainty in *R*, with lower values of *R* associated with higher values of ρ_local_ by 12 March (*SI Appendix*, Fig. S6). Despite this uncertainty due to *R*, the trajectory of ρ_local_ over time was generally robust to alternative parameter scenarios in our sensitivity analysis (*SI Appendix*, Fig. S7). Furthermore, between 24 February (low estimate of ρ_local_) and 12 March, our daily estimates of ρ_local_ in all replicates in our baseline analysis were well correlated with daily numbers of tests administered (Pearson’s correlation, median: 0.98; 95% PPI: 0.66 to 0.98).

**Fig. 3.**
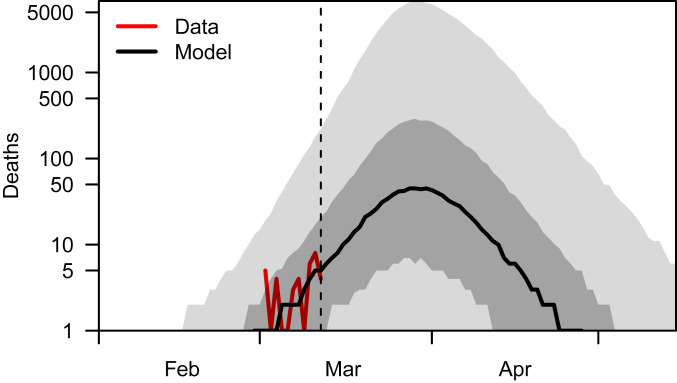
Daily incidence of deaths over time. Our model’s predictions under our baseline analysis were consistent with reported deaths through 12 March (dashed line) and indicate that many more deaths should have been expected after then based solely on infections that occurred by 12 March. This is due to the relatively prolonged delay between infection and death, as well as the model’s prediction that most infections that occurred through 12 March happened fairly close to then. Results to the right of the dashed line do not reflect additional deaths that would result from new infections occurring 13 March or after, which would be expected to add considerably to the number of deaths in April and May. The black line shows the median, dark gray shading shows the interquartile range, and light gray shading shows the 95% PPI.

The fit of our model to cumulative deaths was assessed by its predictions of local deaths by 12 March (median: 35; 95% PPI: 1 to 983), which were consistent with the 39 reported in terms of the central tendency of the model’s predictions (Fig. 3). As with other aspects of our analysis, there was wide uncertainty in our predictions of cumulative deaths, due to stochasticity and parameter uncertainty. Because our model was fit to cumulative deaths only, it was not informed by any information about the timing of those deaths, other than that they occurred by 12 March. Even so, 95.5% of the deaths predicted by our model occurred within the same range of days over which local deaths were reported (29 February to 12 March). This indicates that, collectively, our model’s assumptions about the timing of importation, local transmission, and delay between exposure and death are plausible. Because deaths caused by COVID-19 often occur several weeks after exposure (34), our baseline model predicted a median of 827 (95% PPI: 6 to 115,707) additional deaths after 12 March as a result of infections that occurred by then. Relative to deaths reported by then, this represents an increase by a factor of 21 (95% PPI: 0.18 to 2,965). This result was generally robust in our sensitivity analysis, except that extremely high values of this ratio were ruled out if the serial interval was long or the time to death was short (*SI Appendix*, Fig. S8).

## Discussion

Our approach used a mathematical model to leverage available data to answer a question of significant interest to public health during the initial phase of the COVID-19 pandemic in the United States. The only requirements for applying this approach are basic epidemiological data and estimates of standard epidemiological parameters, both of which are collected routinely during the initial weeks of incipient epidemics. Although other approaches—namely, serological surveys—could have provided more direct answers to the question of how many unobserved infections there were in the weeks following the arrival of SARS-CoV-2 in the United States, serological assays were only beginning to be developed at that time (35). Even now that results from serological surveys are beginning to emerge (36), they still do not address the extent of unobserved infections during the specific time frame of our analysis, are not representative of the United States as a whole, and can be sensitive to even small inaccuracies in assay performance (37, 38). Relative to other approaches, our approach offers the ability to quickly obtain provisional estimates of the number of unobserved infections early in an epidemic, when there still might be time to act on that information with testing and case isolation.

One prominent feature of our results is uncertainty. Although more precise estimates would be preferable, it is important to recognize the inherently uncertain nature of the problem we sought to address. Within the first few months of recognizing a newly emerged pathogen, there is a paucity of data to inform models, considerable uncertainty about parameters related to transmission and disease manifestation, and major unknowns about basic aspects of the pathogen’s biology (39, 40). Our approach offers a way to synthesize multiple aspects of uncertainty from disparate studies to address an important question to which there are no clear answers from more direct, empirical approaches. Some of our results offer more clarity than others. On the one hand, we were unable to resolve much of the uncertainty in two parameters that we estimated in the course of our analysis: ρ_travel_ and α. This was a result of the fact that many different combinations of those parameters were similarly capable of explaining the 39 deaths by 12 March, especially given wide uncertainty on the other parameters that we did not estimate. On the other hand, our results place a high probability (0.825) on the number of local infections in the United States by 12 March being at least an order of magnitude greater than the number of reported cases and quite possibly (with a probability of 0.515) two orders of magnitude or more. Additional precision on our estimate of the number of unobserved infections would not change our overall conclusion that SARS-CoV-2 infections in the United States were severely underreported prior to declaration of a national emergency on 13 March.

In addition to the uncertainty associated with our results, there are other limitations of our analysis that should be acknowledged. First, our branching process model assumes exponential growth, which could be affected by social distancing (41) or the buildup of immunity (42). Neither of those factors seem to have had much influence on local transmission of SARS-CoV-2 in the United States before 13 March, however (43). Second, our parameter assumptions were based primarily on analyses of data collected outside the United States. Similar information has proven useful in past public health emergencies however, such as Zika and Ebola (44, 45), and our accounting for uncertainty about *R*, which is one of the more likely parameters to vary across contexts, spans a wide range of possibilities that could apply to the United States (21). Third, we did not make use of airline data to model importation (46), which could be used to refine assumptions about unobserved importations in future applications of our method (47).

Although the limitations of our analysis limit the precision of our results, we can nonetheless conclude that unobserved SARS-CoV-2 infections in the United States by 12 March could have easily numbered in the hundreds of thousands (31.0% of simulations in our baseline analysis) and quite possibly in excess of 1 million (20.3% of simulations). This result, considered together with extensive presymptomatic and asymptomatic transmission of SARS-CoV-2 (3, 4), suggests that the United States was well past the possibility of containment by 12 March. Other modeling work (16) suggests that the feasibility of containing SARS-CoV-2 is highly sensitive to the number of infections that occur prior to initiation of containment efforts. Our estimate that fewer than 10% of local symptomatic infections were detected by surveillance for around a month is consistent with estimates from a serological study (36) and suggests that a crucial opportunity to limit the impact of SARS-CoV-2 on the United States may have been missed. Although the number of tests administered increased in March (9), so too did the number of infections and consequently, the demand for testing.

Coincident with the 13 March declaration of a national emergency (14), social-distancing measures went into effect across the United States (15). Our estimate of many thousand unobserved SARS-CoV-2 infections at that time suggests that large-scale mitigation efforts, rather than reactionary measures (33), were indeed necessary. Analyses since have indicated that such measures were effective across a wide range of geographic settings (48–51). Even so, acting sooner could have prevented even more cases and deaths (52).

## Materials and Methods

We used a stochastic model, including separate importation and local transmission steps, to represent SARS-CoV-2 transmission in the United States through 12 March 2020. Model parameters and uncertainty therein were either specified according to published studies or calibrated to the total number of deaths reported in the United States through that time period. In our baseline analysis, we used the calibrated model to estimate the total number of SARS-CoV-2 infections in the United States through 12 March, estimate the probability of detecting local symptomatic infections on a daily basis through 12 March, and project the number of deaths expected to occur 13 March and after based only on infections that occurred through 12 March. In our sensitivity analysis, we explored the sensitivity of results from the baseline analysis to alternative choices about parameter values and other assumptions

### Data.

We obtained data on the number of imported cases and deaths from line list data compiled by the Models of Infectious Disease Agent Spread (MIDAS) Network (19). These data informed the number and timing of imported infections predicted by our importation model. We obtained data on the total number of United States cases and deaths and total number of cases and deaths globally from time series compiled by the Johns Hopkins University Center for Systems Science and Engineering (18). These data informed our estimates of the proportion of local infections detected. We also used these data in an alternative importation scenario in which the timing of imported infections was sampled proportional to daily global incidence.

### Model Description.

#### Importation.

We considered cases associated with international travel in the MIDAS dataset to be imported. Due to the fact that SARS-CoV-2–positive individuals who were repatriated from the Diamond Princess cruise ship were quarantined (53), we removed them from our analysis, leaving 153 imported cases (including one death). We estimated the number of imported infections based on the asymptomatic proportion, σ, the case fatality risk (CFR), and the probability of an imported symptomatic infection being detected, ρ_travel_. The probability of the number of unobserved imported infections, *U*, along with the 152 observed cases, *C*, and one observed death, *D*, was calculated as a multinomial probability

$$\text{Pr}\left(\left\{U,C,D\right\}\right)=\frac{\left(U+C+D\right)!}{U!C!D!}\text{Pr}{\left(U\right)}^{U}\text{Pr}{\left(C\right)}^{C}\text{Pr}{\left(D\right)}^{D},$$ [1]

where

$$\mathrm{Pr}\left(U\right)=\sigma +\left(1-\sigma \right)\;\left(1-\rho_{\text{travel}}\right)\;\left(1-\text{CFR}\right)$$ [2a]

$$\text{Pr}\left(C\right)=\left(1-\sigma \right)\;\rho_{\text{travel}}\;\left(1-\text{CFR}\right)$$ [2b]

$$\text{Pr}\left(D\right)=\left(1-\sigma \right)\;\text{CFR}.$$ [2c]

After calculating Pr({*U*,*C*,*D*}) for values of *U* between 0 and 20,000 given fixed values of *C* = 152 and *D* = 1, we used the sample function in R to sample *U* proportional to Pr({*U*,*C*,*D*}). This resulted in posterior samples of *U* conditional on observed values of *C* and *D*. We then smoothed the date of known imported infections with a Gaussian kernel and sampled dates of all *U + C + D* imported infections from that distribution.

#### Local transmission.

We simulated local transmission in the United States from 1 January to 12 March using a branching process model seeded by imported infections. The number of secondary infections generated by each infection in the branching process model was drawn from a negative binomial offspring distribution with mean *R* and dispersion parameter *k*. The number of secondary infections that were generated by asymptomatic individuals was also drawn from a negative binomial distribution but with mean α*R*, where α in [0,1]. Each secondary infection’s exposure time was drawn from a log-normal generation interval distribution. In doing so, we assumed that the generation interval followed the same distribution as the serial interval.

#### Infection outcomes.

In addition to exposure, we simulated three additional outcomes, and the timing thereof, in a subset of infections.

Symptomatic infection: The number of new symptomatic infections on day *t* was drawn from a binomial distribution with the number of trials equal to the number of infections with time of potential symptom onset on day *t* and the probability of success equal to 1 − σ. For infections that resulted in symptoms, the time of symptom onset was drawn from a Weibull distribution and added to each individual’s exposure time.
Potentially reported case: The number of cases that could have potentially been reported on day *t* was drawn from a binomial distribution with the number of trials equal to the number of infections with time of potential case reporting on day *t* and the probability of success equal to 1 − σ. The time of potential case reporting was drawn from a gamma distribution of the period between symptom onset and case reporting and added to each infection’s time of symptom onset.
Death: The number of deaths on day *t* was drawn from a binomial distribution with the number of trials equal to the number of symptomatic infections that could have experienced death on day *t* and the probability of success equal to CFR. The time of death was drawn from a log-normal distribution of time from symptom onset to death and added to each individual’s time of symptom onset.

### Model Parameterization.

All parameter values, and their associated distributions, are described in Table 1. Where parameter distributions were described in the literature using medians and interval measures of spread, we used the optim function in R to estimate parameter values that matched distribution moments reported by those studies. In that sense, all parameters in our analysis were treated as random variables, with associated uncertainty accounted for throughout our analysis. For most parameters, this reflected uncertainty based on an estimate from a single study, given that few studies have generated high-quality estimates for some parameters. In the case of *R*, our estimate was taken from a meta-analysis (21) and thereby, captured uncertainty within and between studies. For the delay between symptom onset and case notification, we fit a gamma distribution to data on the delay between symptoms and reporting for 26 US cases in the MIDAS line list data (19); the gamma distribution fit the data better than negative binomial or log-normal distributions according to the Akaike information criterion (AIC; 133.5, 134.6, and 134.0, respectively) (*SI Appendix*, Fig. S1). Our mean estimate of 6.0 for this delay is in line with previous estimates from China of 5.8 by Li et al. (29) and 5.5 by Bi et al. (24).

Due to a lack of available estimates for ρ_travel_ and α, we estimated them by fitting the model to the total number of deaths resulting from locally acquired SARS-CoV-2 infections in the United States by 12 March. To approximate a likelihood for ρ_travel_ and α, we simulated 200 replicate time series of imported infections, each based on the same value of ρ_travel_, and then simulated local transmission using the same value of α for each of the 200 replicates. For each of these 200 replicate simulations, we calculated the cumulative number of infections, *I*_*D*_, that based on their timing, could have resulted in death by 12 March. We then calculated the likelihood as the probability of the reported number of deaths, *D*, according to a binomial distribution in which *D* ∼ Binomial(*I*_*D*_, CFR (1 − σ)). To account for the effect of stochasticity on *I*_*D*_, we estimated the marginal likelihood of ρ_travel_ and α over a grid of values between 0 (or 0.01 for ρ_travel_) and 1 in increments of 0.05 for each parameter. At each point on this grid, 200 replicate likelihoods were obtained, meaning that our estimate of the marginal likelihood of ρ_travel_ and α was informed by a total of 88,200 simulations. We represented this marginal likelihood surface with a generalized additive model (GAM) fit to these 88,200 likelihoods using thin-plate splines with 100 knots using the mgcv package (54) in R. We selected 100 knots based on AIC and chose this over default settings of the gam function due to the sensitivity of the latter to noise in the likelihoods, as indicated by its prediction of multiple peaks in the marginal likelihood surface. We then used this GAM to create a gridded marginal likelihood surface with a 0.001 × 0.001 mesh. Finally, we drew samples from the posterior probability distribution of these parameters by sampling with replacement over this mesh, which implicitly assumed a uniform prior on ρ_travel_ and α.

### Baseline Analysis.

Under the baseline parameter values in Table 1, we used the calibrated model to perform three analyses.

First, we generated a distributional estimate of the total number of SARS-CoV-2 infections in the United States through 12 March, based on 1,000 realizations of the model. Each realization drew independent values of each parameter, such that the distributional estimate of SARS-CoV-2 infections reflected both parameter uncertainty and stochasticity inherent to the earliest stage of the epidemic. This stochasticity included randomness in the number and timing of imported infections, as each simulation used a separate realization of the importation process to seed local transmission.

Second, we estimated how the probability of detecting local symptomatic infections, ρ_local_, changed over time. These estimates were based on the number of symptomatic cases reported each day, *C*(*t*), and our model’s predictions of the number of symptomatic infections that could have been reported each day, *S*(*t*), after accounting for a delay between symptom onset and reporting. We assumed a uniform prior for ρ_local_ and on each day, estimated a posterior equal to ρ_local_(*t*) ∼ Beta(1 + *C*(*t*), 1 + *S*(*t*) − *C*(*t*)). We then took 1,000 replicates of independent daily draws of logit-transformed values of ρ_local_(*t*) and smoothed over them using the smooth.spline function in the stats package in R, using weekly knots (*SI Appendix*, Fig. S2).

Third, we estimated how many deaths would occur 13 March and after based only on infections that occurred through 12 March. The purpose of this was to illustrate the effect of the relatively long lag between infection and death on patterns of death over time. To do this, we simulated 1,000 realizations of our model through 12 March and then set *R* = 0 from 13 March to 31 May. This allowed infections occurring by 12 March enough time to result in death.

### Sensitivity Analysis.

We undertook a one-at-a-time sensitivity analysis for each parameter shown in Table 1, with the exception of ρ_travel_, α, and *R*. Including the baseline analysis, this resulted in a total of 16 scenarios (i.e., the baseline plus low and high values for each of seven parameters plus 1 alternative scenario for importation timing). For each scenario, we repeated each of the three steps in our baseline analysis. We excluded *R* from the sensitivity analysis because between-study variability was already accounted for in our baseline analysis. In the case of ρ_travel_ and α, these parameters were recalibrated for each alternative scenario considered in the sensitivity analysis. For the delay in reporting following symptom onset, we obtained low and high values by multiplying the shape parameter by 0.5 and 1.5, respectively, while holding the rate parameter constant. In this way, this delay is the sum of one, two, or three identically distributed gamma random variables in the low, baseline, and high scenarios, respectively. For importation timing, our alternative scenario involved sampling dates of imported infections based on the timing of international incidence. This excluded cases in China after 3 February, due to a ban on entrance by nonresident foreign nationals who had been to China within the past 14 d enacted on 2 February.

## Supplementary Material

Supplementary File

## Data Availability

All code and data used are available at GitHub (https://github.com/TAlexPerkins/sarscov2_unobserved).
