# Supplementary material for: Estimating unobserved SARS-CoV-2 infections in the United States
Source: Proc Natl Acad Sci U S A. 2020 Aug 21;117(36):22597–602. doi: 10.1073/pnas.2005476117 (PMC7486725; doi:10.1073/pnas.2005476117)
Supplement: Supplementary File [file pnas.2005476117.sapp.pdf]

PNAS
www.pnas.org

Supplementary Information for

Estimating unobserved SARS-CoV-2 infections in the United States

T. Alex Perkins*†, Sean M. Cavany†, Sean M. Moore†, Rachel J. Oidtman, Anita Lerch, Marya Poterek

Department of Biological Sciences and Eck Institute for Global Health, University of Notre Dame

† Equal contributions

* Corresponding author: T. Alex Perkins

Email: taperkins@nd.edu

This PDF file includes:

Supplementary text
Figures S1 to S8
Tables S1 to S3

www.pnas.org/cgi/doi/10.1073/pnas.2005476117

Supplementary Information Text

Imported infection predictions and estimates of imported case detection probability
By March 12, there were a total of 152 reported cases and one reported death in the US that were classified as imported on the basis of international travel to areas with known SARS-CoV-2 transmission (19). By jointly estimating ρ_{travel} and the relative infectiousness of asymptomatic infections, α, we obtained a median estimate of 0.44 (95% PPI: 0.04 - 0.97) for ρ_{travel} under our baseline scenario. This resulted in a median of 621 (95% PPI: 271 – 5,558) imported infections. Under the alternative importation timing scenario, where importation timing was based on international case reports, we obtained a median estimate of 0.56 (95% PPI: 0.10 – 0.97) for ρ_{travel} and 482 (95% PPI: 263 – 2,301) imported infections. An estimate of ρ_{travel} = 1.00 implies that all symptomatic imported infections were detected, but it still means that asymptomatic infections would have been undetected. Whereas the baseline importation scenario resulted in most importations happening in March and a few throughout February and January, the alternative importation scenario resulted in many importations still happening in March but a large proportion of them also happening around late January (Fig. S3).

Posterior predictive check against reported local cases
Using our estimate of $\rho_{local}(t)$, we simulated the number of reported cases through time and compared this with the actual number of reported cases. By March 12, our model predicted that there should have been 1,014 (95% PPI: 102 - 4,593) reported cases, commensurate with the actual number of 1,514 reported cases (Fig. 2B). As expected, this confirms that our estimates of $\rho_{local}(t)$ were consistent with the model and the data.

Sensitivity analysis of unknown parameters
Estimates of the proportion of imported symptomatic infections that were detected, ρ_{travel}, and the infectiousness of asymptomatic infections relative to symptomatic infections, α, varied based on the values of the other parameters. In general, higher values for parameters expected to increase or accelerate transmission (e.g., a shorter serial interval) were associated with higher estimates of ρ_{travel} (Table S1). For a shorter mean serial interval of 4.7 days, the estimate was ρ_{travel} = 0.50 (95% PPI: 0.05 - 0.97), and with a longer mean serial interval of 7.5 days, the estimate was 0.13 (95% PPI: 0.01 - 0.79). The estimated value of ρ_{travel} was also lower if the CFR was low (ρ_{travel} = 0.40, 95% PPI: 0.03 - 0.96), compared to the scenario with a higher CFR (ρ_{travel} = 0.55, 95% PPI: 0.06 - 0.98). Higher ρ_{travel} estimates correspond to fewer undetected imported infections; therefore, fewer undetected importations are required to account for the observed number of local deaths through March 12 if the CFR is high or the serial interval is short. In addition, when we based the timing of importations on international incidence (excluding China after travel restrictions were implemented on February 3) the estimate of ρ_{travel} was 0.56 (95% PPI: 0.10 - 0.97) due to the increased probability of early importations – and more time for local infections to increase – under this scenario. In most scenarios, the estimates of ρ_{travel} and α were positively correlated (Fig. S4).

Sensitivity analysis of cumulative infections
Because ρ_{travel} and α were estimated for each parameter-sensitivity scenario, cumulative infections were relatively similar under the low, baseline, and high scenarios for nearly all parameters. Cumulative infections were most sensitive to assumptions about the serial interval (Fig. S5, Table S2). This affects how quickly local infections increase. Cumulative infections were also somewhat sensitive to assumptions about case fatality risk, because assumptions about that parameter influenced estimates of ρ_{travel} and α, which were based on reported deaths.

Sensitivity analysis of local case detection probability
The proportion of symptomatic infections detected over time followed a similar pattern under all parameter sensitivity scenarios, with low values of $\rho_{local}(t)$ throughout late February followed by increases in March (Fig. S7).

Sensitivity analysis of the ratio of deaths after and before March 12

The ratio of deaths expected March 13 and after, relative to before then, was higher with changes in parameters that resulted in faster growth in local infections (e.g., shorter serial intervals) (Fig. S8, Table S3). The proportion of deaths expected to occur after March 12 also increased with shorter reporting delays (Table S3). Overdispersion (lower *k*) did not drastically alter our estimates of ρ_{travel} or α (Table S1) or the number of cumulative infections (Table S2), but it did extend the lower and upper bounds on the range of the ratio of deaths after and before March 12 (Table S3).

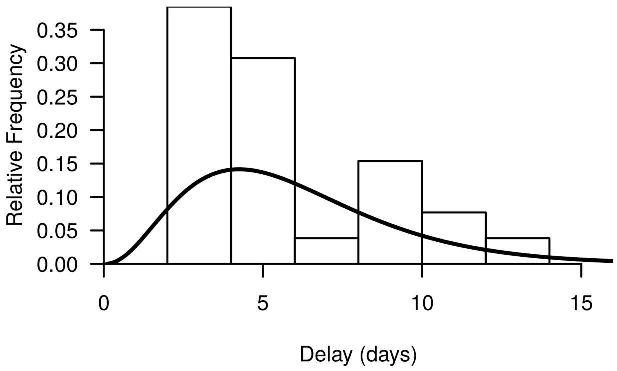

Figure S1. Distribution of the delay between symptom onset and reporting for 26 US cases. The curve shows the maximum-likelihood fit of a gamma distribution (shape = 3.43, rate = 0.572) to those data.

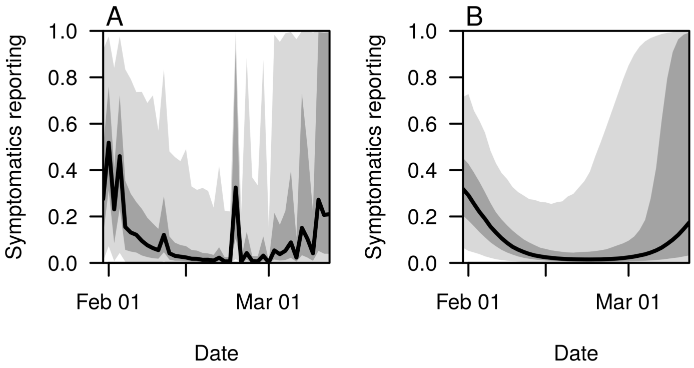

Figure S2. Comparison of daily estimates of $\rho_{local}(t)$ with and without smoothing in the baseline analysis. The two panels compare A) raw estimates with no smoothing and B) smoothed estimates with splines. We used the smoothed estimates in our analysis given that they are more indicative of general trends in case detection. The black line shows the median, dark gray shading shows the interquartile range, and light gray shading shows the 95% posterior predictive interval.

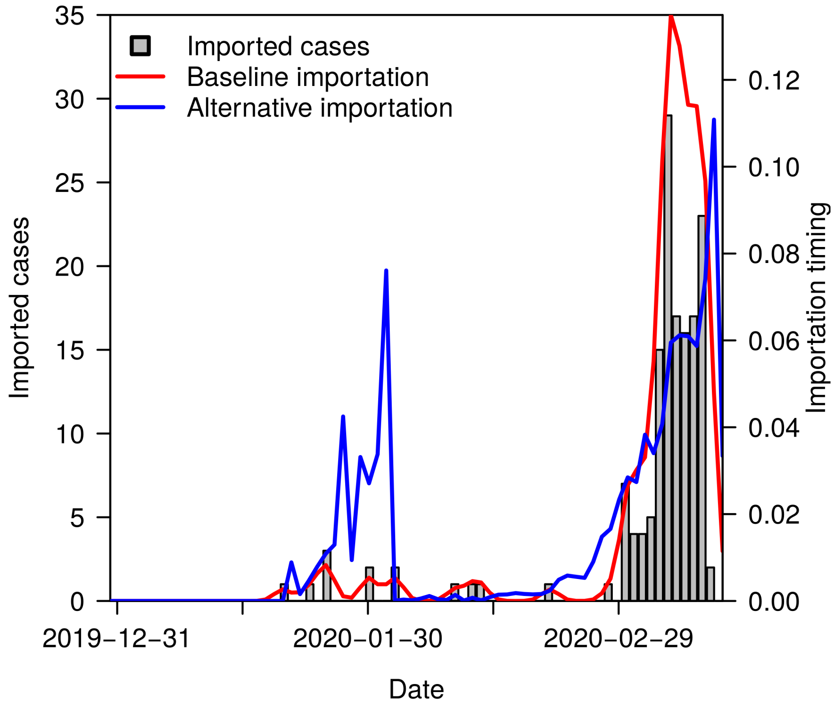

Figure S3. Assumptions about timing of imported infections. Imported cases that have been reported are shown in gray, and the red line shows the baseline distribution of timing of imported infections that we based on a Gaussian kernel smooth of those data. The blue line shows an alternative distribution of timing of imported infections based on patterns of international incidence.

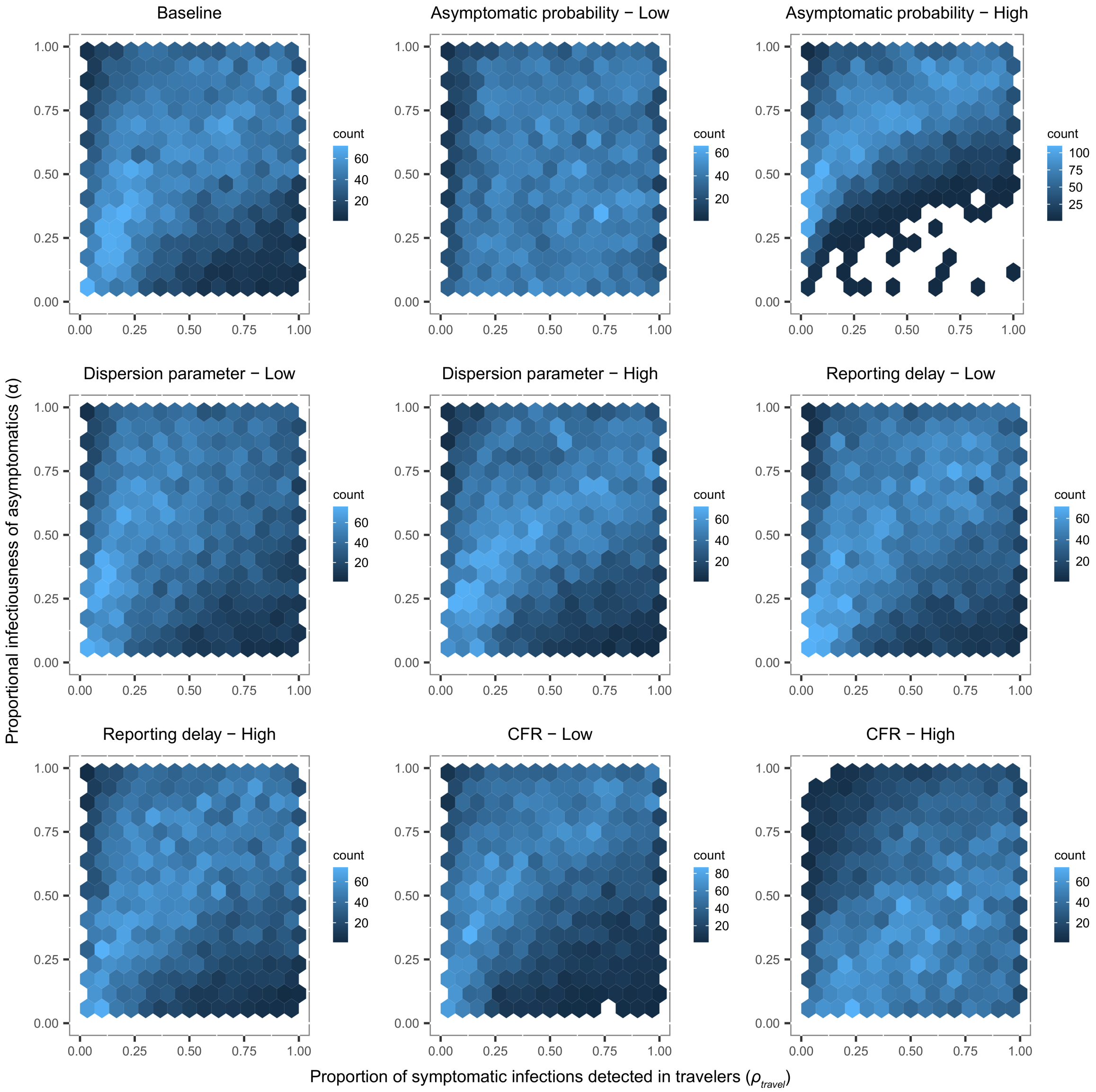

Figure S4. Samples (10^4) from the joint posterior distribution of the proportion of imported symptomatic infections detected (ρ_{travel}) and the relative infectiousness of asymptomatic infections (α) under different parameter-sensitivity scenarios. Parameter values for these scenarios are shown in Table S1 (for ρ_{travel} and α) and Table 1 (for all other parameters).

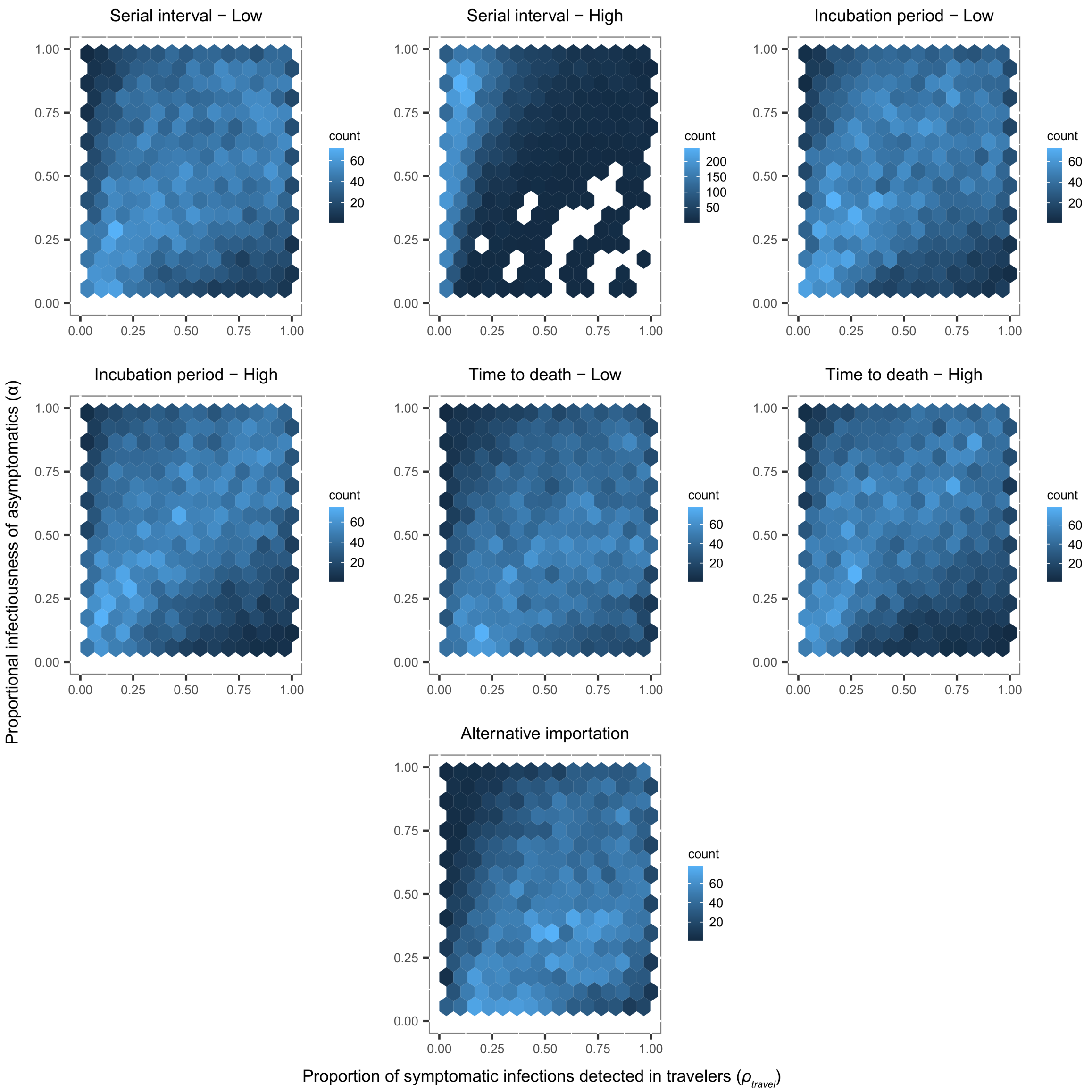

Figure S4 (continued). Samples (10^4) from the joint posterior distribution of the proportion of imported symptomatic infections detected (ρ_{travel}) and the relative infectiousness of asymptomatic infections (α) under different parameter-sensitivity scenarios. Parameter values for these scenarios are shown in Table S1 (for ρ_{travel} and α) and Table 1 (for all other parameters).

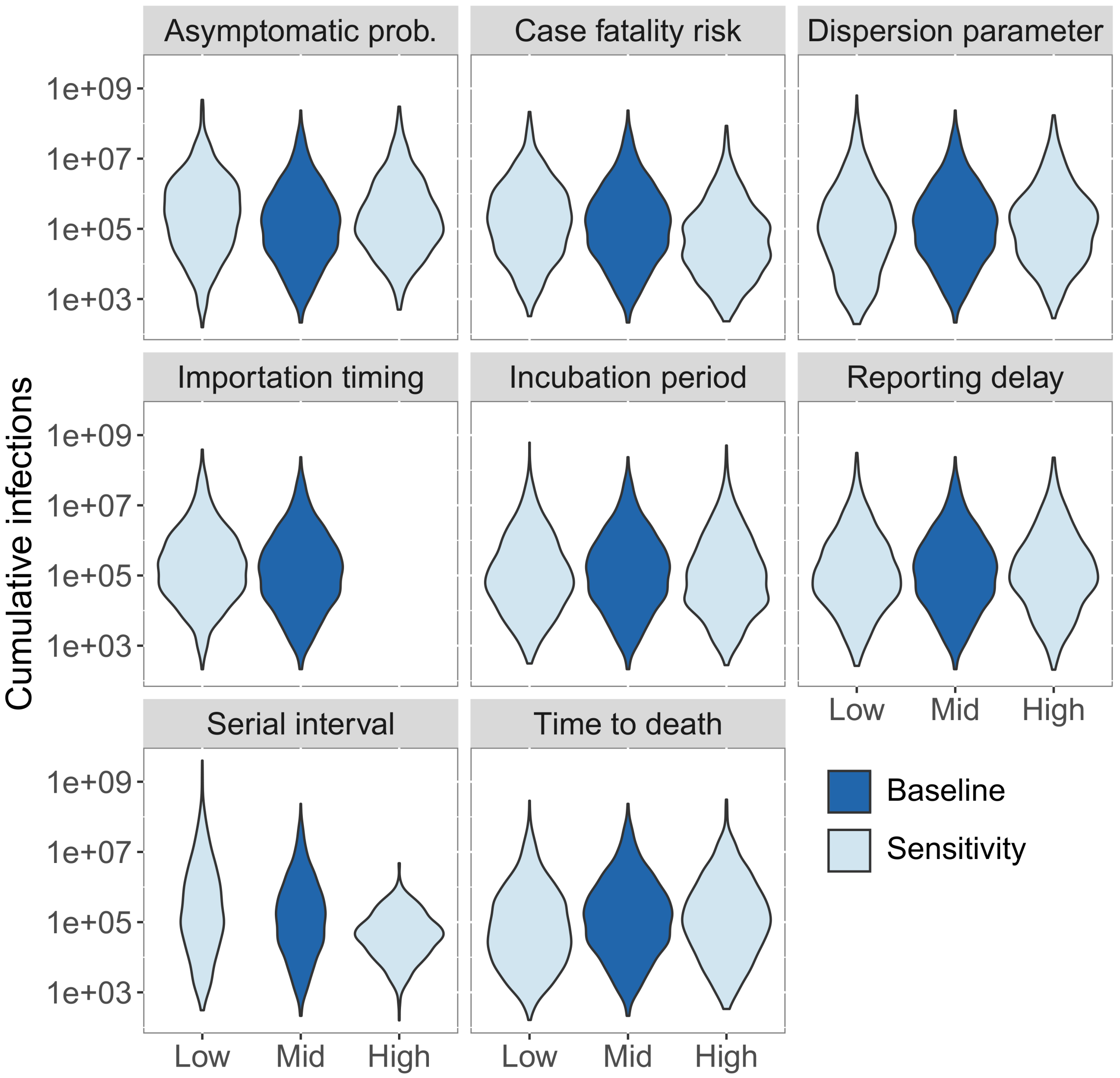

Figure S5. Posterior predictive distributions of cumulative infections by March 12 under different parameter sensitivity scenarios. Unlike other parameters, importation timing was not described in terms of simple numerical values; in that case, "mid" refers to our baseline assumption that the timing of unobserved imported infections followed the timing of observed imported cases, and "high" refers to the alternative scenario that their timing followed international incidence patterns. Parameter values for these scenarios are shown in Table S1 (for ρ_{travel} and α) and Table 1 (for all other parameters).

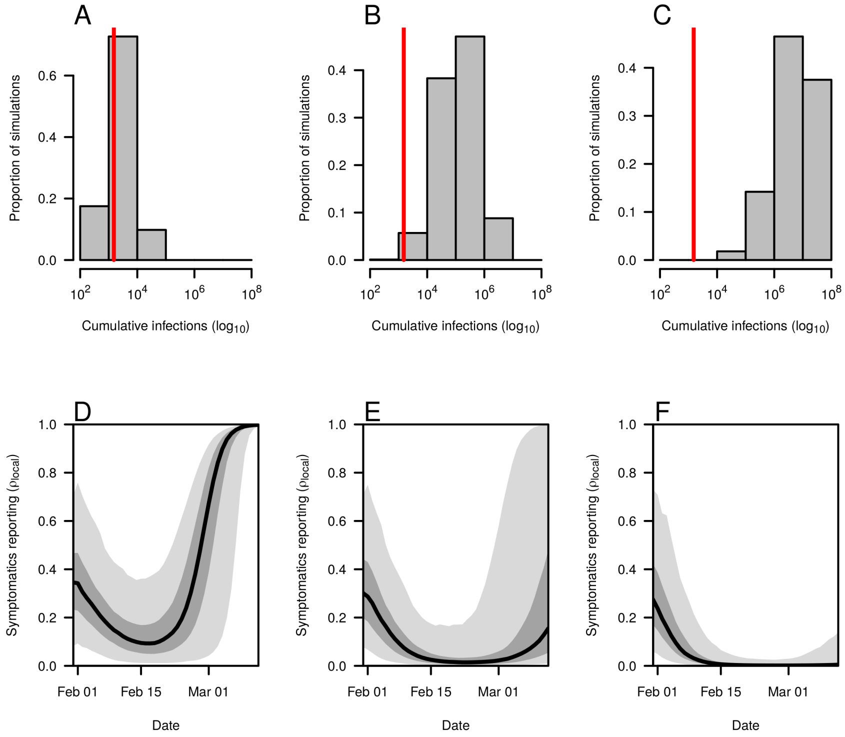

Figure S6. A-C) Posterior predictive distributions of cumulative infections by March 12 from the baseline analysis, with each panel reflecting a fixed value of *R*: A) 1.9, B) 2.7, C) 3.9. D-F) Median (black), interquartile range (dark gray), and 95% posterior predictive interval (light gray) of the probability of detecting a local symptomatic infection, $\rho_{local}(t)$, from the baseline analysis, with each panel reflecting a fixed value of *R*: D) 1.9, E) 2.7, F) 3.9. For the other parameters, 1,000 draws were made from the uncertainty distributions corresponding to the baseline analysis in Table S1 (for ρ_{travel} and α) and Table 1 (for all other parameters).

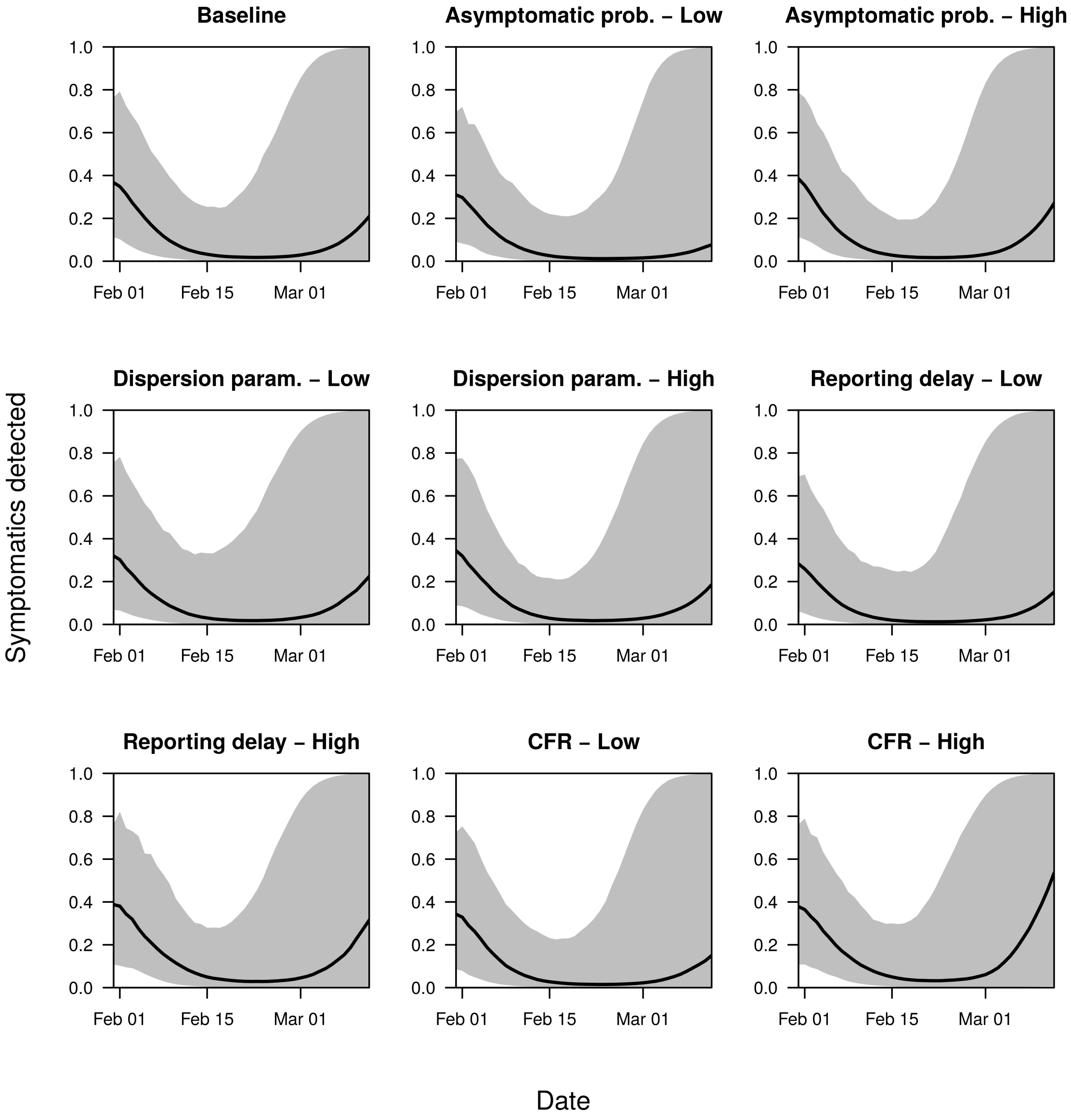

Figure S7. Median (black) and 95% posterior predictive interval (gray) of the probability of detecting a local symptomatic infection, $\rho_{local}(t)$, after accounting for delays in reporting. Each panel represents a different parameter-sensitivity scenario. Parameter values for these scenarios are shown in Table S1 (for ρ_{travel} and α) and Table 1 (for all other parameters).

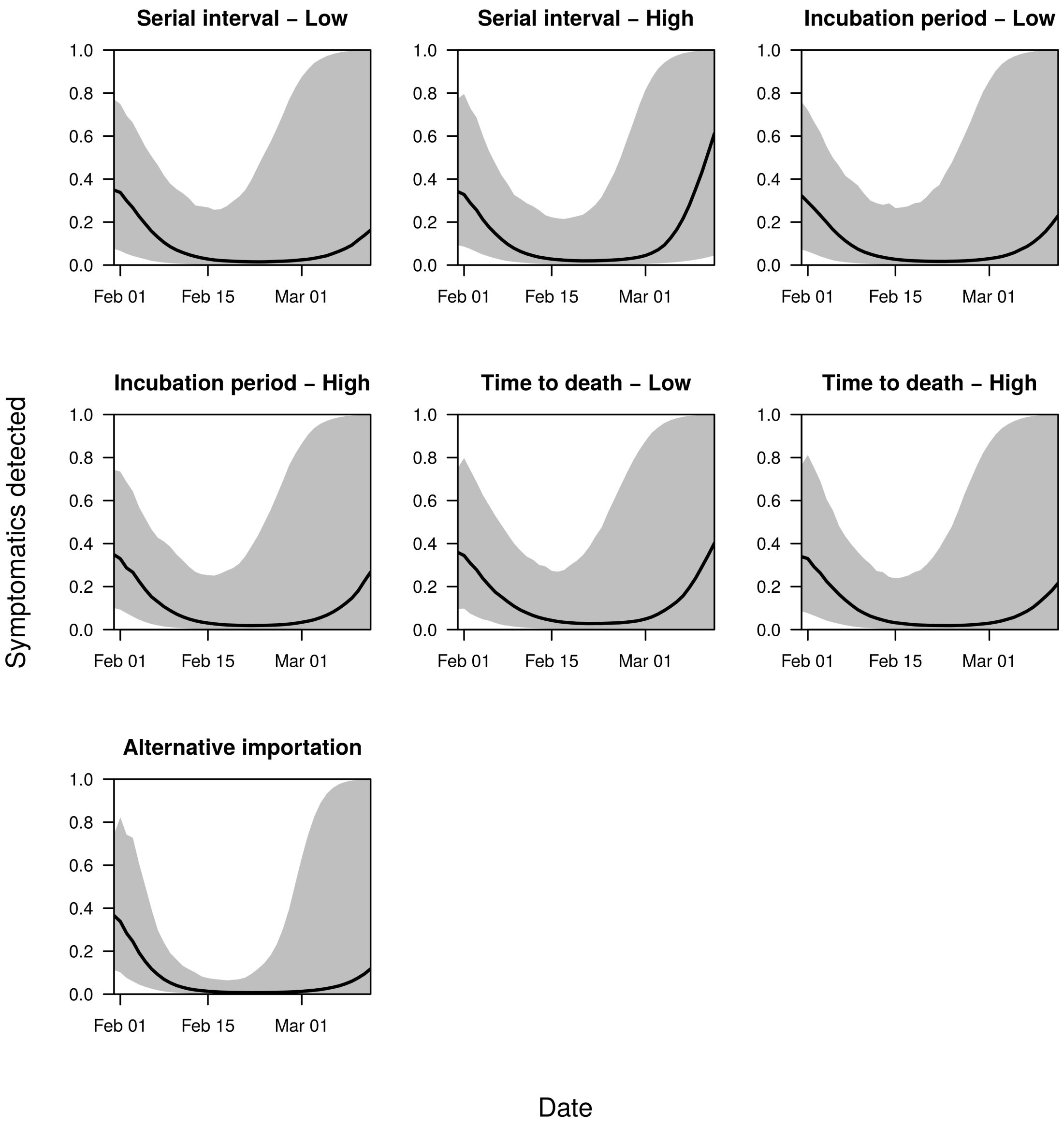

Figure S7 (continued). Median (black) and 95% posterior predictive interval (gray) of the probability of detecting a local symptomatic infection, $\rho_{local}(t)$, after accounting for delays in reporting. Each panel represents a different parameter-sensitivity scenario. Parameter values for these scenarios are shown in Table S1 (for ρ_{travel} and α) and Table 1 (for all other parameters).

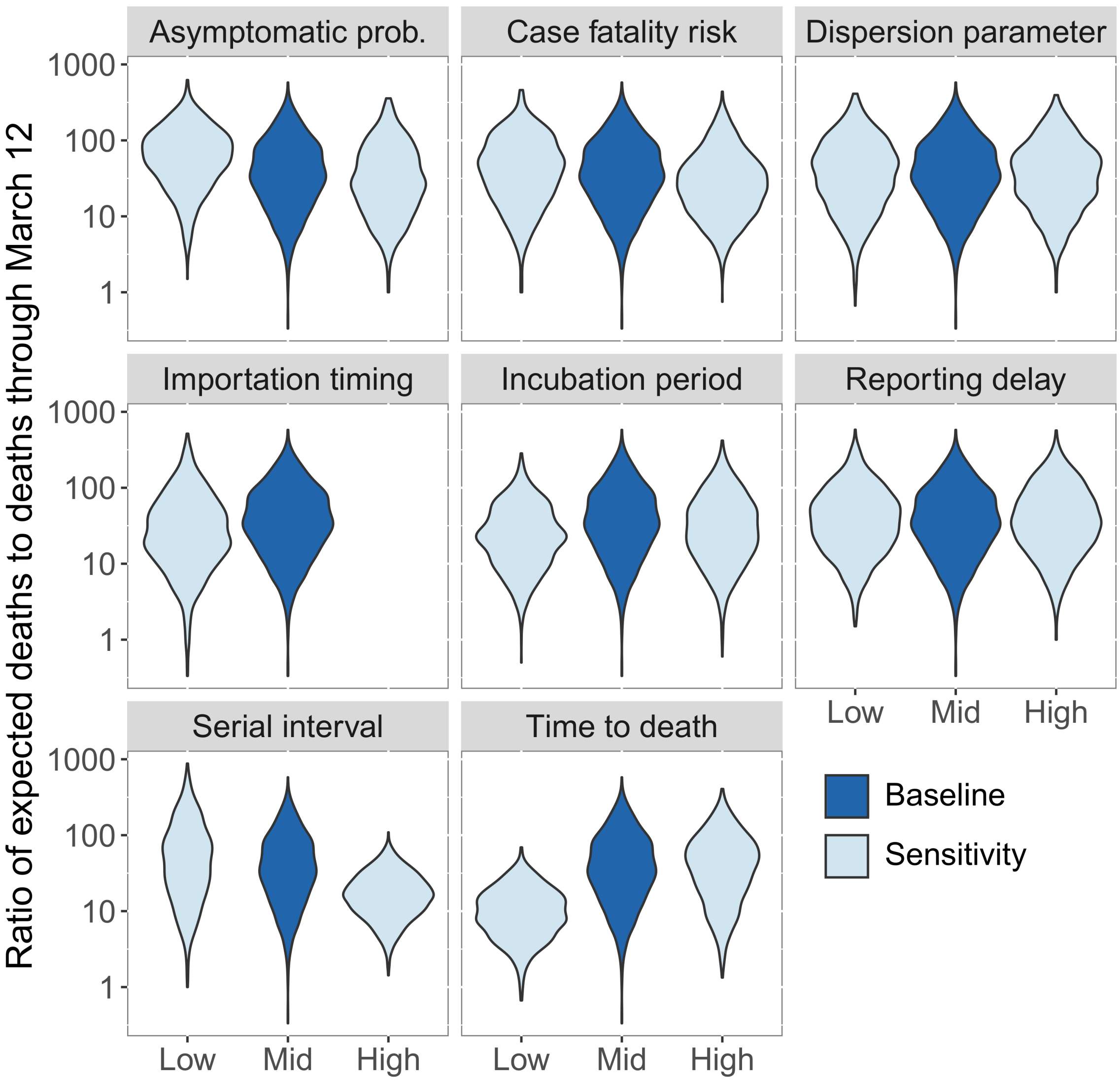

Figure S8. Posterior predictive distributions of the ratio of deaths after and before March 12 under different parameter sensitivity scenarios. Unlike other parameters, importation timing was not described in terms of simple numerical values; in that case, “mid” refers to our baseline assumption that the timing of unobserved imported infections followed the timing of observed imported cases, and “high” refers to the alternative scenario that their timing followed international incidence patterns. Parameter values for these scenarios are shown in Table S1 (for ρ_{travel} and α) and Table 1 (for all other parameters).

Table S1. Median estimates and 95% posterior predictive intervals of the marginal distributions of proportion of imported symptomatic infections detected (ρ_{travel}) and the relative infectiousness of asymptomatic infections (α) under different parameter-sensitivity scenarios.

**Parameter varied**	**Scenario**	**Prob. symptomatic infections detected (ρ_{travel})**			**Relative asymptomatic infectiousness (α)**		
		median	2.5%	97.5%	median	2.5%	97.5%
Baseline		0.438	0.041	0.969	0.553	0.046	0.973
Asymptomatic prop.	Low	0.537	0.073	0.974	0.481	0.022	0.971
Asymptomatic prop.	High	0.407	0.016	0.964	0.716	0.266	0.984
Dispersion, *k*	Low	0.405	0.034	0.963	0.55	0.042	0.976
Dispersion, *k*	High	0.449	0.038	0.970	0.542	0.050	0.973
Reporting delay	Low	0.443	0.037	0.969	0.55	0.047	0.974
Reporting delay	High	0.444	0.04	0.971	0.558	0.047	0.978
CFR	Low	0.403	0.027	0.963	0.606	0.062	0.979
CFR	High	0.548	0.062	0.976	0.406	0.02	0.948
Serial interval	Low	0.495	0.046	0.973	0.492	0.031	0.970
Serial interval	High	0.127	0.007	0.792	0.705	0.079	0.988
Incubation period	Low	0.46	0.044	0.967	0.5165	0.04	0.971
Incubation period	High	0.462	0.045	0.969	0.534	0.04	0.972
Time to death	Low	0.522	0.063	0.976	0.449	0.026	0.961
Time to death	High	0.448	0.037	0.969	0.551	0.043	0.975
Importation timing	High	0.555	0.095	0.974	0.422	0.025	0.958

Table S2. Median estimates and 95% posterior predictive intervals of cumulative infections under different parameter sensitivity scenarios.

**Parameter varied**	**Scenario**	**Cumulative infections (95% PPI)**		
		median	2.5%	97.5%
Baseline		129,696	1,128	18,940,383
Asymptomatic prop.	Low	255,904	1,161	25,969,364
Asymptomatic prop.	High	170,567	2,910	29,202,284
Dispersion, *k*	Low	99,613	613	24,566,833
Dispersion, *k*	High	120,974	1,201	19,223,968
Reporting delay	Low	103,544	1,020	19,194,774
Reporting delay	High	112,822	1,049	21,893,968
CFR	Low	172,374	1,406	17,245,335
CFR	High	41,970	615	6,244,867
Serial interval	Low	174,506	1,119	73,856,319
Serial interval	High	41,660	1,860	614,836
Incubation period	Low	87,990	1,108	15,936,532
Incubation period	High	82,543	1,162	17,110,240
Time to death	Low	56,596	869	12,436,371
Time to death	High	118,196	1,069	16,727,194
Importation timing	International incidence	174,278	1,665	29,303,022

Table S3. Median estimates and 95% posterior predictive intervals of the ratio of deaths after and before March 12 under different parameter sensitivity scenarios.

**Parameter varied**	**Scenario**	**Ratio of future deaths to death by March 12**		
Baseline		35.7	3.8	214.6
Asymptomatic prop.	Low	61.6	6.7	261.3
Asymptomatic prop.	High	27.4	3.5	207.6
Dispersion, *k*	Low	35.9	3.2	220.4
Dispersion, *k*	High	34.5	4.1	221.4
Reporting delay	Low	36.4	4.4	219.1
Reporting delay	High	35.8	4.3	228.9
CFR	Low	42	3.5	211.1
CFR	High	24.9	4.3	178.5
Serial interval	Low	45.4	4.2	343.7
Serial interval	High	15.4	3.8	52.7
Incubation period	Low	22.2	3.8	119.1
Incubation period	High	29.2	3.8	179.5
Time to death	Low	9.5	1.8	34.9
Time to death	High	37.9	3.8	200.1
Importation timing	International incidence	24.4	2.6	213.8
